# Chronic administration of plasma from exercised rats to sedentary rats does not induce redox and metabolic adaptations

**DOI:** 10.1186/s12576-020-00737-2

**Published:** 2020-02-03

**Authors:** Georgios Goutianos, Nikos V. Margaritelis, Theodora Sparopoulou, Aristidis S. Veskoukis, Ioannis S. Vrabas, Vassilis Paschalis, Michalis G. Nikolaidis, Antonios Kyparos

**Affiliations:** 1 Department of Physical Education and Sport Science at Serres, Aristotle University of Thessaloniki, Agios Ioannis, 62110 Serres, Greece; 2 Intensive Care Unit, 424 General Military Hospital of Thessaloniki, Thessaloniki, Greece; 3 Department of Animal Structure and Function, School of Veterinary Medicine, Faculty of Health Sciences, Aristotle University of Thessaloniki, Thessaloniki, Greece; 4 Department of Biochemistry and Biotechnology, University of Thessaly, Larissa, Greece; 5 School of Physical Education and Sport Science, National and Kapodistrian University of Athens, Athens, Greece

**Keywords:** Adaptations, Exercise, Muscle, Plasma injection, Rats, Redox

## Abstract

The present study aimed to investigate whether endurance exercise-induced changes in blood plasma composition may lead to adaptations in erythrocytes, skeletal muscle and liver. Forty sedentary rats were randomly distributed into two groups: a group that was injected with pooled plasma from rats that swam until exhaustion and a group that was injected with the pooled plasma from resting rats (intravenous administration at a dose of 2 mL/kg body weight for 21 days). Total antioxidant capacity, malondialdehyde and protein carbonyls were higher in the plasma collected from the exercised rats compared to the plasma from the resting rats. Νo significant difference was found in blood and tissue redox biomarkers and in tissue metabolic markers between rats that received the “exercised” or the “non-exercised” plasma (*P* > 0.05). Our results demonstrate that plasma injections from exercised rats to sedentary rats do not induce redox or metabolic adaptations in erythrocytes, skeletal muscle and liver.

## Introduction

Blood “composition” dramatically changes during and a few hours after exercise. The blood levels of some molecules increase in response to acute exercise (e.g., inflammatory cytokines), while the levels of other molecules decrease (e.g., myostatin). Diverse metabolically active tissues throughout the human body, such as the liver, skeletal muscle and adipose tissue, exert significant endocrine activity affecting distal organs, contributing thereby to the altered chemical composition of blood [1, 2]. Myokines and adipokines (i.e., cytokines released from skeletal muscle and adipose tissue, respectively) are two representative examples of molecules that are released into the bloodstream during and after exercise [1, 2]. Along with these tissues, there is compelling evidence supporting an active role of blood (predominantly via its cells) as a source of bioactive molecules that mediate the signals for biochemical and physiological adaptations in other tissues and organs [3, 4]. Even the blood plasma, which is widely considered an inert body fluid that receives metabolic by-products of other tissues, has been recently reported to act as the intermediary “modifier” niche for tissue-originated circulating molecules [5]. Thus, the role of blood as a transporter, producer and modifier of bioactive molecules seems to be of particular importance when investigating exercise adaptations and other physiological and biochemical phenotypes, as well.

This is best exemplified by the elegant experimental approaches implemented by different research groups in order to reveal the role of circulating molecules in diverse biological phenomena (e.g., exercise adaptations, healthy aging, longevity). These approaches include the incubation of cells in mediums containing either components or the whole secretome of other cells [6, 7], cell or tissue cultures incubated with serum from exercised [8–11] or calorie-restricted individuals [12–15], transplantation of white fat from exercised to sedentary animals [16], parabiosis set-ups between transgenic and wild-type exercised animals [17], as well as between young and aged animals [18–25], plasma injection from exercised to sedentary rats [26] and from young to aged animals [25] and, finally, execution of isolated body part or limb exercise protocols [27–32]. According to the available data, there is some evidence suggesting that changes in circulating molecules can stimulate the production of factors that subsequently affect other tissues. However, with regard to exercise, a great debate exists in the literature about the role of post-exercise increases in several humoral factors on skeletal muscle adaptations (e.g., anabolism and hypertrophy) [33].

All the aforementioned experimental designs provide valuable information on the role of blood or its constituents, however, each design has some fundamental limitations [e.g., in vitro to in vivo extrapolation (cell culture studies), use of highly invasive techniques (parabiosis studies) and parallel effect of the neural system (isolated body part exercise studies)]. Plasma injection in rodents, although not without limitations, seems to be a rather non-invasive and effective model to study in vivo the effect of circulating factors in tissue and organ adaptations [26, 34]. Regarding exercise, and to the best of our knowledge, only one study has used this experimental approach and has reported that plasma injection from exercised rats to sedentary rats induced systemic and tissue inflammation [(i.e., interleukins, tumor necrosis factor alpha (TNF-α) and C-reactive protein (CRP)] [26]. This may also have important implications in the redox homeostasis of the sedentary rats, since inflammatory and redox processes are strongly interrelated. Noteworthy, it has been recently demonstrated in vivo that post-exercise oxidative stress is a key factor in endurance training adaptations [35], while the fundamental nature of redox biology of exercise is increasingly recognized [36–39]. Thus, the aim of the present study was to investigate the effect of “exercised” plasma injection in mediating systemic and tissue redox and metabolic exercise adaptations in sedentary rats, mimicking the impact of whole-body endurance exercise.

## Materials and methods

### Animals

Adult male Wistar rats, weighing 380 ± 27 g (mean ± SD) were used in the study. Rats were housed under a 12 h light:12 h dark cycle, controlled temperature (21–23 °C) and humidity (50–70%). Commercial rat chow and tap water were provided ad libitum. All procedures were in accordance with the European Union guidelines for the care and use of laboratory animals, as well as the “Principles of laboratory animal care” (NIH publication No. 86-23, revised 1985). The project was reviewed and approved by the institutional review board and the appropriate state authority (#359888/3612).

### Experimental design

**Fig. 1:**
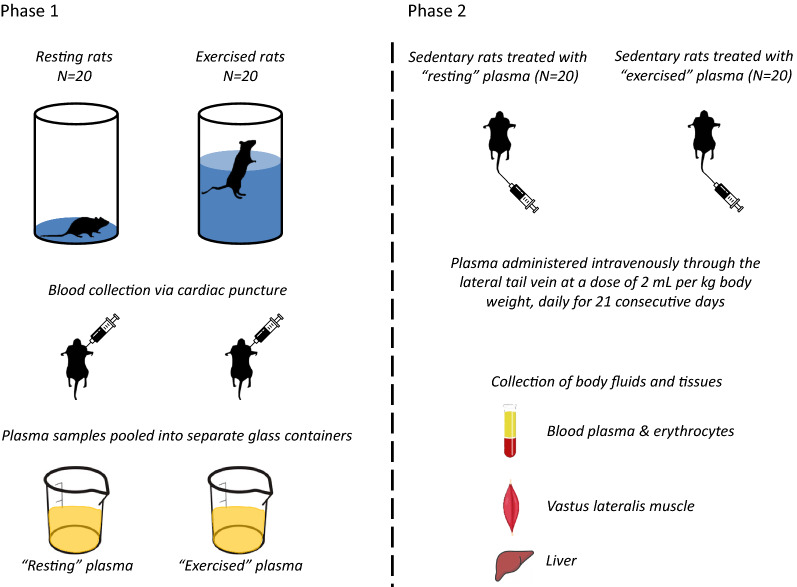
The study design

The whole study design is shown in Fig. 1.

#### Phase 1

Blood samples were collected from: (i) exercised rats immediately after a swimming bout to exhaustion and (ii) from resting rats. Following centrifugation, blood plasma was separated from blood cells. The plasma samples were pooled into two separate glass containers and were homogenized (one container included the plasma from all the exercised rats and one container included the plasma from all the resting rats). Subsequently, the pooled plasma mixtures were put into aliquots of 0.8 mL and stored in plastic tubes at − 80 °C for use in phase 2 and later analysis.

### Phase 2

Forty (*N* = 40) sedentary rats were randomly distributed into two groups as follows: (i) a group that was injected with the pooled plasma collected from the exercised rats that swam until exhaustion in phase 1 (*N* = 20) and (ii) a group that was injected with the pooled plasma collected from the resting rats of phase 1 (*N* = 20). Injection of either the exercised or resting plasma was administered intravenously through the lateral tail vein at a dose of 2 mL per kg body weight, daily for 21 consecutive days. The duration of the administration process was set based on the intention to mimic the exercise-induced changes in plasma for a long time period, while the dose was selected so that each administration would not induce great acute changes in blood volume of rats (approximately 3.5% volume was added).

In very few aging studies on cognitive function, the injection dose intravenously into the mice tail vein was 100 μL, 4 times over 10 days [24], or 100 μL, 8 times over 24 days [25]. The present study is the first attempt in the field of exercise physiology to incorporate plasma injections from exercised rats to sedentary counterparts. An adequate number of acute “exercise bouts” should be performed to induce chronic adaptation response. It was assumed that a 3-week “exercised” plasma administration period could simulate sufficiently the shortest, but adequate, “training period” for exercise adaptations to occur and detected.

The tail vein injections were performed in both lateral tail veins starting from the tip of the tail and gradually moving towards the base of the tail, using 1 mL insulin syringes, rat restrainers and a tail vein injection platform. Twenty-four hours following the last injection, all animals were killed, samples of blood plasma and erythrocyte lysate, vastus lateralis muscle and liver were collected and stored at − 80 °C for later analysis.

### Swimming familiarization and exercise protocol—phase 1

Rats were familiarized with swimming for 4 consecutive days [26]. The daily swimming duration was 10 min with various loads attached on the base of their tails, ranging from 0% to approximately 4% of their body weight. In particular, rats swam with no load on day 1 (first contact with the water tanks), while the adjusted loads were 5 g, 10 g and 15 g for days 2, 3 and 4, respectively. Following the 4-day familiarization protocol, the rats were allowed to rest for 48 h prior the exhaustive swimming bout.

The swimming protocol for the exercised rats was performed in plastic cylindrical tanks (diameter: 1.2 m, height: 1.1 m, water depth: 0.7 m in order to prevent rats from jumping out of the tank and from touching the bottom of the tanks with their tails). Water temperature was maintained between 33.5 and 34.5 °C. Rats swam individually in the tank and the swimming effort was gradually increased by addition of loads attached to their tails. In particular, after an initial weight of 5 g in all rats for the first 15 min of exercise, the load was then gradually increased by 5 g every 5 min until exhaustion. Animals were considered to have reached exhaustion when they exhibited loss of coordinated movements and inability to return to the surface within 10 s for three consecutive times [26]. On the other hand, the resting rats were placed in the swimming tank containing only a minimal amount of water to wet their limbs, for a time period equal to the average swimming time to exhaustion of the exercised rats.

### Blood and tissue collection and preparation for analysis

Rats were deeply anesthetized as described previously [26]. Then, the thoracic cavity was opened and whole blood was collected via cardiac puncture of the right ventricle using a 10-mL syringe (Terumo, Tokyo, Japan) in vacutainer tubes containing no additives (for phase 1) or ethylenediaminetetraacetic acid (EDTA) (for phase 2) (BD Vacutainer Systems, Plymouth, U.K.). Whole blood samples were immediately centrifuged (1500*g*, 4 °C, 10 min) for separation of plasma from blood cells. After plasma collection, the remaining supernatant in the EDTA tubes (i.e., plasma residue, platelets and white blood cells) was discarded. An equal volume to the packed erythrocytes of distilled water was added to the tubes, the samples were centrifuged (4000*g*, 15 min, 4 °C) and the supernatant hemolysate (i.e., red blood cell lysate) was collected. The erythrocyte hemolysate was then stored at − 80 °C for later analysis.

Immediately after blood sampling, the vastus lateralis muscle (VL) and the liver were rapidly removed, snap frozen in liquid nitrogen and stored at − 80 °C for later analysis. To grind the tissue samples for analysis, a mortar and pestle under liquid nitrogen were used. Tissue powder was then homogenized (1:2 w/v ratio) with 10 mmol/L phosphate-buffered saline (PBS) (138 mmol/L NaCl, 2.7 mmol/L KCl, and 1 mmol/L EDTA, pH = 7.4) and a cocktail of protease inhibitors (1 μmol/L aprotinin, 100 μmol/L leupeptin and 1 mmol/L phenylmethylsulfonyl fluoride) to block proteolytic cleavage of proteins. The homogenate was vigorously vortexed, briefly sonicated on ice and centrifuged (12,000 g, 4 °C, 30 min). The supernatant was collected and stored at − 80 °C for subsequent analysis.

### Biochemical assays

The following measurements were performed: total antioxidant capacity in plasma and vastus lateralis muscle; malondialdehyde in plasma; protein carbonyls in plasma and vastus lateralis muscle; catalase, superoxide dismutase and glutathione reductase activity in erythrocytes and vastus lateralis muscle; reduced glutathione content in erythrocytes and vastus lateralis muscle; citrate synthase activity in vastus lateralis muscle; glycogen content in vastus lateralis muscle and in liver.

Citrate synthase activity was measured in vastus lateralis muscle as previously described [40]. Glycogen concentration was measured in vastus lateralis muscle and liver via a modified protocol of Lo et al. [41] and Hoshino et al. [42] and was subsequently calculated with the use of a standard curve created based on known glycogen concentrations. Total antioxidant capacity in blood plasma and vastus lateralis muscle was measured according to a protocol described previously [43]. Plasma malondialdehyde concentration was measured based on Keles et al. [44] and Lapenna et al. [45] and calculated with the use of the molar extinction coefficient of malondialdehyde. Catalase, superoxide dismutase and glutathione reductase activity as well as the content of protein carbonyls and glutathione were measured as previously described by Veskoukis et al. [46]. Results were normalized to total protein for plasma, vastus lateralis muscle and liver and normalized to hemoglobin for erythrocytes. Total protein content was measured using the Bradford assay via a standard curve of solutions with known bovine serum albumin concentrations. Hemoglobin concentration was measured spectrophotometrically using the cyanmethemoglobin method with a commercial kit (Hemoglobin liquicolor, Human, Wiesbaden, Germany) according to manufacturer’s instructions. All biochemical variables were determined spectrophotometrically.

### Statistical analysis

**Fig. 2:**
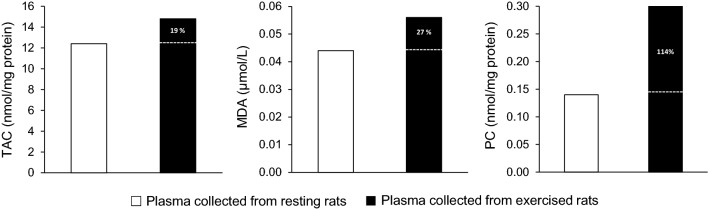
Redox biomarkers in pooled plasma samples collected either from resting (open bars) or exercised (closed bars) rats. Percent values indicate the relative change of exercised compared to resting values. The two pooled plasma samples were treated as two single treatments; thus, no inferential statistics could be performed. *TAC* total antioxidant capacity, *MDA* malondialdehyde, *PC* protein carbonyls

Independent samples Student’s t‐tests (SPSS Inc., Chicago, IL; version 21) were used to compare the dependent variables measured in blood plasma, erythrocytes, vastus lateralis and liver in the two experimental groups of the phase 2 (i.e., the groups that were injected with the pooled plasma collected either from the exercised or the resting rats). The pooled plasma samples (exercised and resting) of the phase 1 were treated as two single samples. As a result, no standard deviation could be computed and no inferential statistics were performed (Fig. 2). The significance level was set at *P* < 0.05. Data are presented as mean ± standard deviation (SD).

## Results

### Phase 1

The exercising group of rats swam until exhaustion for 28.9 ± 4.5 min. The average load during the swimming protocol (attached on the base of each rat’s tail) was equal to 2.62 ± 0.55% body weight. Resting rats were placed in empty swimming tanks containing only a minimal amount of water to wet their limbs for 29 min, in order to match the time period of the swimming protocol of the exercised rats.

The level of total antioxidant capacity (TAC), malondialdehyde (MDA) and protein carbonyls (PC) was numerically higher in the pooled plasma collected from the exercised rats compared to the pooled plasma collected from the resting rats by 19% (TAC, 12.4 vs. 14.8 nmol/mg protein), 27% (MDA 0.044 vs. 0.056 μmol/L) and 114% (PC 0.14 vs. 0.30 nmol/mg protein), respectively (no inferential statistics performed) (Fig. 2). The two pooled plasma samples were subsequently used as the experimental treatments in phase 2.

### Phase 2

#### Effects of blood plasma injection on redox biomarkers

**Fig. 3:**
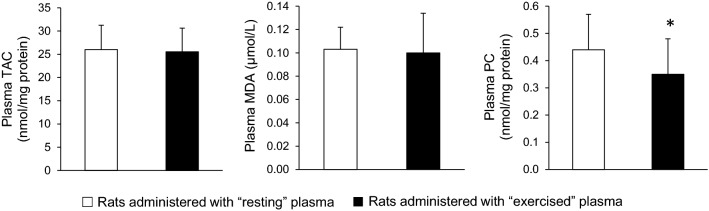
Redox biomarkers in plasma of sedentary rats following plasma administration of either resting (open bars) or exercised (closed bars) rats. *TAC* total antioxidant capacity, *MDA* malondialdehyde, *PC* protein carbonyls. (*) indicates significant difference between groups

In blood plasma, no significant differences were found in total antioxidant capacity (26.0 ± 5.3 vs. 25.5 ± 5.1 nmol DPPH/mg protein) and malondialdehyde (0.10 ± 0.02 vs. 0.10 ± 0.03 μM) between the group that received the plasma from the resting rats and the group that received the plasma from the exercised rats (*P* > 0.05). However, a significant difference was found between these groups in plasma protein carbonyls (0.44 ± 0.13 vs. 0.35 ± 0.13 nmol/mg protein, respectively) (Fig. 3).

**Fig. 4:**
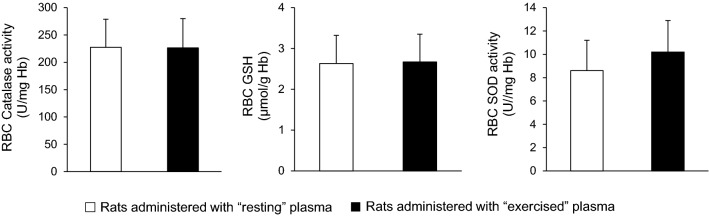
Antioxidants in red blood cells of sedentary rats following plasma administration of either resting (open bars) or exercised (closed bars) rats. *CAT* catalase, *GSH* reduced glutathione, *SOD* superoxide dismutase

In erythrocyte lysate, no significant differences were found in glutathione (2.63 ± 0.69 vs. 2.67 ± 0.88 μmol/g Hb), catalase activity (227 ± 51 vs. 226 ± 53 U/mg Hb) and superoxide dismutase activity (8.60 ± 2.61 and 10.2 ± 2.71 U/mg Hb) between the group that received the plasma from the resting rats and the group that received the plasma from the exercised rats (*P* > 0.05) (Fig. 4). Only a trend toward significance in superoxide dismutase activity was observed (*P* = 0.065).

**Fig. 5:**
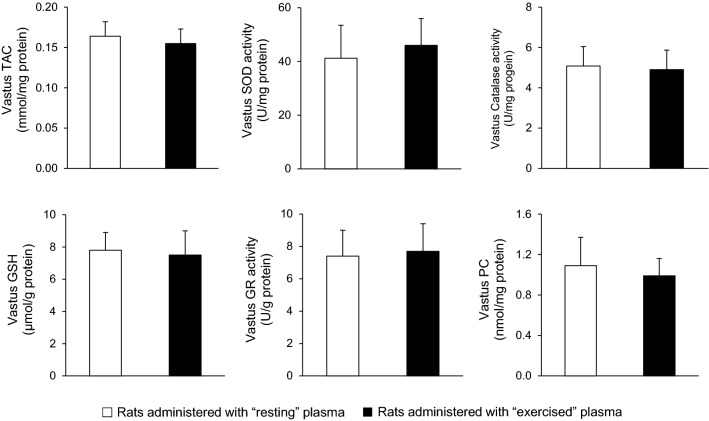
Redox biomarkers in vastus lateralis muscle of sedentary rats following plasma administration of either resting (open bars) or exercised (closed bars) rats. *TAC* total antioxidant capacity, *SOD* superoxide dismutase, *CAT* catalase, *GSH* reduced glutathione, *GR* glutathione reductase, *PC* protein carbonyls

In vastus lateralis muscle, no significant differences were found in total antioxidant capacity (164 ± 18 vs. 155 ± 18 μmol DPPH/mg protein), glutathione (7.80 ± 1.12 vs. 7.51 ± 1.52 μmol/g protein), protein carbonyls (1.09 ± 0.28 vs. 0.99 ± 0.17 nmol/mg protein), catalase activity (5.08 ± 0.97 vs. 4.90 ± 0.97 U/mg protein), superoxide dismutase activity (41.2 ± 12.3 vs. 46 ± 10 U/mg protein) and glutathione reductase activity (7.42 ± 1.63 U/g vs. 7.74 ± 1.71 U/g protein) between the group that received the plasma from the resting rats and the group that received the plasma from the exercised rats (*P* > 0.05) (Fig. 5).

#### Effects of blood plasma injection on tissue metabolic adaptation biomarkers

**Fig. 6:**
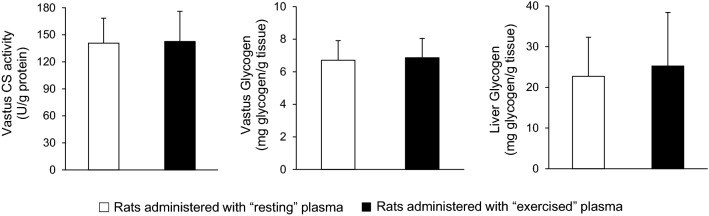
Metabolic training adaptation biomarkers in vastus lateralis muscle and liver of sedentary rats following plasma administration of either resting (open bars) or exercised (closed bars) rats. *CS* citrate synthase

In vastus lateralis muscle, no significant differences were found in citrate synthase activity (140.8 ± 27.6 vs. 142.6 ± 33.5 U/g protein) and glycogen concentration (6.71 ± 1.20 vs. 6.86 ± 1.19 mg glycogen/g tissue) between the group that received the plasma from the resting rats and the group that received the plasma from the exercised rats (*P* > 0.05) (Fig. 6). No significant difference was also found in liver glycogen concentration (22.7 ± 9.6 vs. 25.2 ± 13.2 mg glycogen/g tissue) between the two groups (*P* > 0.05) (Fig. 6).

## Discussion

Rodents are a good model to study the effects of exercise on various physiological systems and conditions [47–55]. Acute exercise dramatically alters blood composition. Blood is the recipient of secretomes originated in other tissues with endocrine properties, such as skeletal muscle and liver, while the blood itself also produces bioactive molecules [3, 4]. Mounting evidence suggests that the transient enrichment of blood biochemical “cocktail” in response to exercise facilitates the signal transmission to target cells and organs. Diverse methodological set-ups have been implemented in order to reveal the role of blood in exercise adaptations [8–11, 16, 17, 27–29]. In the present study, we have used plasma injections, an in vivo approach that has never been applied previously in an exercise setting and does not require largely invasive manipulations. In particular, for 21 days we injected to sedentary rats (phase 2), plasma previously collected from other rats (phase 1) that either swam until exhaustion or rested (control group) for a matching time period (i.e., 29 min). Therefore, different rats of similar age and weight were used in phase 1 and phase 2. In phase 1, the individual plasma values of the examined variables were not measured. Rather, the plasma from all animals (separately in resting or exercised groups) were pooled and mixed in two containers. Then, the variables were measured in the pooled plasma of each group.

We aimed thereby to investigate whether the transient exercise-induced changes in circulating plasma molecules, contribute chronically to classic endurance training-induced metabolic adaptations in other tissues (i.e., skeletal muscle and liver glycogen content and muscle citrate synthase activity) [56, 57]. Although numerous factors have been characterized as signals for adaptations (i.e., hormones, cytokines), we particularly focused on redox molecules (i.e., enzymatic and non-enzymatic antioxidants and oxidative stress biomarkers), since redox processes are nowadays considered an essential component of exercise metabolism [36–39].

According to our findings, there was an increase in plasma redox and oxidative stress biomarkers after exhaustive swimming in phase 1 (TAC 19%, MDA 27%, PC 114%). However, this typical increase in redox “content” of pooled “exercised” plasma was not a sufficient stimulus to induce redox and/or metabolic adaptations in the rats received this plasma in phase 2. We herein report that the chronic plasma injection collected from exercised rats did not affect redox status in erythrocytes and vastus lateralis muscle of sedentary rats, since none of the biomarkers has changed.

Regarding plasma measurements, the treatment partially affected oxidative stress biomarkers, as suggested by the reduction in the plasma protein carbonyl concentration. This finding seems, at a first sight, as unexpected, since the administration of “exercised” plasma was anticipated to increase concentration of this abundant and chemically stable oxidative stress biomarker. However, it has been recently demonstrated that plasma protein carbonyl content is determined by the dynamic balance between the reactive species-induced production of protein carbonyl groups and their clearance by the 20S proteasome system [58]. This is also in line with the general idea that the oxidation products should be considered neither as end-products nor as inactive molecules [59]. Regarding tissue exercise metabolic adaptations, the injection of plasma collected from exercised rats did not affect any metabolic biomarker in vastus lateralis muscle (citrate synthase activity and glycogen content) or liver (glycogen content).

The general idea of our study was that the repetitive injections of plasma from exercised rats to sedentary rats would replicate the “episodic” pattern of exercise training and would alter, at least in part, the circulating milieu, mimicking thereby the effects of whole-body exercise. In other words, we considered plasma injection as a more physiological exercise “mimetic” approach compared to diverse natural or synthetic drugs that have been developed and aim to replicate the metabolic and physiological effects of exercise (the “exercise in a pill” theory; [60, 61]. The lack of an effect in our study could be attributed to several reasons. It is possible that some of the exercise-induced plasma factors that were injected to sedentary rats could not transport across the cell membrane into the tissue (e.g., through transporters activated during exercise) and, as a result, no tissue effect was observed. Even in the case of humoral factors that can permeate the cell membrane (e.g., via diffusion), it is plausible to suggest that the presence of these molecules per se is not sufficient to trigger the sequence of molecular events needed for training adaptations. In fact, multiple molecular (activation of transcription factors), biochemical (redox reactions), metabolic (changes in AMP/ATP ratio), biomechanical (shear stress) and physiological (intracellular hypoxia) changes, inherent to each individual tissue, take place transiently during exercise [62, 63]. All these parameters seem to be essential for training adaptations to occur and, thus, the isolated contribution of blood plasma is not satisfactory to induce adaptations. Finally, our results may also indicate that the endurance training tissue adaptations are primarily driven by local (e.g., intramuscular) processes and not by humoral factors. This issue has been a matter of debate lately in a comparable context, that is, skeletal muscle anabolism and hypertrophy. In particular, controversy exists regarding the contribution of circulating anabolic hormones and growth factors in exercise-induced muscle hypertrophy, with the muscle-centric theory being currently considered as the prevailing theory [33].

Our findings are partially in contrast with other studies that underlined the central role of blood in exercise adaptations and other biological processes as well (e.g., aging). There are several explanations for these differences. First, we injected blood plasma, thus, factors present only in this particular body fluid were transferred. Bioactive molecules present in or originated from blood cells were inevitably excluded. In this context, the promising results from parabiosis set-ups, which facilitate the transfer of whole blood from one organism to another (e.g., between a young and an aged animal) by sharing a common circulatory system, may stem from blood cell-derived and not plasma factors [20, 21, 24, 25]. Especially regarding endurance training, several humoral factors (e.g., catecholamines, peptides and hormones) can adjust the hematopoietic process, upregulating red blood cell production and volume, which subsequently can improve maximal oxygen uptake [64, 65]. Secondly, we used a combined ex vivo/in vivo setting, which is by definition highly dynamic, a fact that could explain the different results compared to studies that incubated cells in mediums containing the secretome of other cells or in serum from different athletes [6–8, 10, 11]. In particular, the rats that received the plasma (in phase 2) from the exercised or resting rats (from phase 1), as any biological system, may have “responded” initially to the exogenous stimulus (i.e., plasma injection) and became subsequently “unresponsive” to the specific treatment (plasma injection of 2 mL per kg body weight). Thirdly, the role of blood in regulating exercise adaptations may share some, but not all, mechanisms with other biological conditions, such as aging [18, 19, 24, 25] or calorie restriction [12–15]. This could be a key reason why the positive outcomes reported previously in these situations (i.e., aging and calorie restriction) were not substantiated in our exercise study.

Certainly, some limitations have to be acknowledged. Perhaps, ideally, recipient sedentary animals should have received the blood plasma from progressively trained animals (from day 1 to day 21) to more closely mimic the chronic exercise adaptation. However, such an experiment would have required a series of parallel experiments and a large number of animals. With regard to redox biomarkers, there was not a complete panel of measurements in all specimens (i.e., plasma, erythrocytes and vastus lateralis muscle) due to sampling and analytical issues. In particular, malondialdehyde was measured only in plasma, glutathione reductase activity was measured only in vastus lateralis muscle, while protein carbonyls were not measured in erythrocyte lysate. Glutathione concentration and the activity of the antioxidant enzymes (i.e., catalase and superoxide dismutase) were purposively measured only in erythrocytes and skeletal muscle, but not in plasma, due to vague biological interpretation (i.e., compartmentalization of redox processes; [66]). On the other hand, a recent study underlined the usefulness of redox enzyme measurements in plasma in an exercise context providing a nuanced view on their applicability [67]. In addition, our redox measurements consisted only of oxidative stress biomarkers and antioxidant molecules. Despite the fact that these measurements are necessary to pinpoint likely redox components in a physiological process [68], we did not include any mechanistic redox biomarker, such as a redox-sensitive transcription factor that relates to exercise adaptations [e.g., nuclear factor erythroid 2-related factor 2 (Nrf2) or nuclear factor kappa-light-chain-enhancer of activated B cells (NF-κB)], to acquire a mechanistic perspective, as well. It is increasingly recognized that in order to more tightly integrate redox signaling events into biological processes, such as exercise adaptations, mechanistic measurements are essential [69]. Another limitation is that we did not assess any physiological (e.g., muscle function, contractile properties) or performance (time trial, fatigue test) endpoint along with the redox and metabolic measurements. Such endpoints would have enhanced the translational potential of our study. Finally, all outcome measures in phase 2 were performed under resting/basal conditions and it is likely that the results would differ in response to a physiological challenge (e.g., differences in citrate synthase and antioxidant enzymes activity).

## Conclusion

In the present study, we applied a plasma injection set-up to examine the role of plasma circulating factors on systemic and tissue redox and metabolic training-induced adaptations. Most of the evidence presented herein demonstrates that repetitive daily injections of plasma from exercised rats to sedentary rats did not induce any redox or metabolic adaptation in the erythrocytes, vastus lateralis muscle and liver. These results indicate that endurance training adaptations rely predominantly on tissue- or blood cell-specific processes and highlight the fact that exercise induces an orchestrated response that necessitates both humoral factors and cell preparation.

## Data Availability

Not applicable.

## Abbreviations

CRP: C-reactive protein
DPPH: 2,2-Diphenyl-1-picrylhydrazyl
EDTA: Ethylenediaminetetraacetic acid
Hb: Hemoglobin
MDA: Malondialdehyde
PBS: Phosphate-buffered saline
PC: Protein carbonyls
TAC: Total antioxidant capacity
TNF-α: Tumor necrosis factor alpha
VL: Vastus lateralis muscle
